# SLE Peripheral Blood B Cell, T Cell and Myeloid Cell Transcriptomes Display Unique Profiles and Each Subset Contributes to the Interferon Signature

**DOI:** 10.1371/journal.pone.0067003

**Published:** 2013-06-24

**Authors:** Amy M. Becker, Kathryn H. Dao, Bobby Kwanghoon Han, Roger Kornu, Shuchi Lakhanpal, Angela B. Mobley, Quan-Zhen Li, Yun Lian, Tianfu Wu, Andreas M. Reimold, Nancy J. Olsen, David R. Karp, Fatema Z. Chowdhury, J. David Farrar, Anne B. Satterthwaite, Chandra Mohan, Peter E. Lipsky, Edward K. Wakeland, Laurie S. Davis

**Affiliations:** 1 Department of Immunology, The University of Texas Southwestern Medical Center, Dallas, Texas, United States of America; 2 Department of Internal Medicine, The University of Texas Southwestern Medical Center, Dallas, Texas, United States of America; 3 Autoimmunity Branch, National Institute of Arthritis and Musculoskeletal and Skin Diseases, National Institutes of Health, Bethesda, Maryland, United States of America; Pavillon Kirmisson, France

## Abstract

Systemic lupus erythematosus (SLE) is a chronic autoimmune disease that is characterized by defective immune tolerance combined with immune cell hyperactivity resulting in the production of pathogenic autoantibodies. Previous gene expression studies employing whole blood or peripheral blood mononuclear cells (PBMC) have demonstrated that a majority of patients with active disease have increased expression of type I interferon (IFN) inducible transcripts known as the IFN signature. The goal of the current study was to assess the gene expression profiles of isolated leukocyte subsets obtained from SLE patients. Subsets including CD19^+^ B lymphocytes, CD3^+^CD4^+^ T lymphocytes and CD33^+^ myeloid cells were simultaneously sorted from PBMC. The SLE transcriptomes were assessed for differentially expressed genes as compared to healthy controls. SLE CD33^+^ myeloid cells exhibited the greatest number of differentially expressed genes at 208 transcripts, SLE B cells expressed 174 transcripts and SLE CD3^+^CD4^+^ T cells expressed 92 transcripts. Only 4.4% (21) of the 474 total transcripts, many associated with the IFN signature, were shared by all three subsets. Transcriptional profiles translated into increased protein expression for CD38, CD63, CD107a and CD169. Moreover, these studies demonstrated that both SLE lymphoid and myeloid subsets expressed elevated transcripts for cytosolic RNA and DNA sensors and downstream effectors mediating IFN and cytokine production. Prolonged upregulation of nucleic acid sensing pathways could modulate immune effector functions and initiate or contribute to the systemic inflammation observed in SLE.

## Introduction

Systemic lupus erythematosus (SLE) is an autoimmune disease that involves activation of both the innate and adaptive immune response. SLE occurs as a result of a disturbance in the homeostatic maintenance of immune tolerance, likely initiated by a pathogen challenge, combined with genetic defects that contribute to subsequent autoimmune hyperactivity [1]. SLE can cause a number of symptoms including nephritis, inflammation of the nervous system, arthritis, leukopenia and skin rashes [2]. The organ-specific nature of the disease appears to be driven by several distinct elements including pathogenic autoantibodies, immune complexes, T cell activation in the absence of sufficient regulation, apoptotic cells and local factors contributed by target tissues. Moreover, recent studies indicate that the presence of autoantibodies formed after breaks in immune tolerance to self-antigens likely result in the initiation of end organ disease [1], [3]–[5].

Dysregulation of type I interferons (IFN) has been implicated in the pathogenesis of SLE [6]–[9]. IFN was elevated in serum samples from SLE patients and correlated with SLEDAI scores and other clinical parameters of disease. The finding that some patients treated with IFN therapy developed autoantibodies and IFN drives B cell differentiation and autoantibody production suggest a mechanistic role for IFN in disease pathogenesis [5], [10], [11]. Both an IFN signature and a granulopoeisis signature have been identified in SLE PBMC or whole blood samples [12]–[16]. However, these studies have not delineated the contribution of each major leukocyte subset to the differentially regulated transcripts observed in SLE.

This study addressed the above knowledge gap by focusing on select PBMC subsets from SLE patients. PBMC are composed of several cell types, including monocytes, dendritic cells, natural killer cells, B and T lymphocytes. The findings highlight the unique nature of the transcriptional profiles in CD19^+^CD3^−^ B cells, CD3^+^CD4^+^ T cells and CD33^+^CD3^−^ myeloid cells, and raise the possibility that the implicated transcriptional networks might be important in disease pathogenesis. Furthermore, our results suggest that the increased transcriptional activity in SLE leukocytes translates into elevated protein expression. Thus the proteins reflected by the gene expression profiles, such as those observed to be upregulated in the nucleic acid sensing pathways, could contribute to the propagation of systemic inflammation.

## Materials and Methods

### Study Participants

The study protocol was approved by the Institutional Review Board of the University of Texas Southwestern Medical Center (UTSW). After obtaining written informed consent, phlebotomy was carried out on SLE patients and healthy controls (HC). All SLE patients fulfilled at least 4 of 11 American College of Rheumatology classification criteria for SLE. Disease activity assessed at the time of blood acquisition was calculated using the systemic lupus erythematosus disease activity index (SLEDAI) score as we have previously described [17]. For the arrays, we compared PBMC subsets from a total of fifteen female SLE patients (mean age 39±12 years) and eleven female HC (mean age 37±10 years). Although patients were on a variety of disease modifying agents, patients on high dose immunocytotoxic therapies or steroids were excluded from the study. However, patients on lower doses of prednisone (10–20 mg/day; and 1 patient on 40 mg/day) were included. Patient characteristics for samples assessed in the arrays are shown in Table 1.

**Table 1:** SLE patient characteristics.

Patient	Age	SLEDAI	dsDNA	C3/C4	ESR	Prot	Medications
14	28	22	Increased	Low	115	Yes	Pred
13	21	12	Increased	Low	70	No	None
12	38	9	Normal	Normal	nd	No	None
11	44	8	Increased	Low	70	No	HCQ, Pred
10	35	12	Normal	Low	nd	Yes	MM, Pred
09	41	14	Increased	Low	>130	No	AZP, Pred
08	57	4	Normal	Normal	67	Yes	HCQ, Pred
07	46	8	Increased	Low	115	No	MTX, Pred
06	27	2	Normal	Normal	22	No	HCQ, Pred
05	57	4	Normal	Normal	65	Yes	HCQ, AZP
04	32	7	Increased	Low	32	No	HCQ, Pred
03	55	7	Normal	Normal	106	Yes	MTX
02	21	10	Increased	Normal	22	Yes	HCQ, Pred
01	42	4	Normal	Normal	35	Yes	None

Patient characteristics and disease activity (SLEDAI) scores for samples analyzed by microarray. All patients were female. dsDNA indicates anti-dsDNA antibodies, reference range <50 IU/ml was Normal, >60 IU/ml was Increased; C3, normal values were 0.75–1.65 g/l, C4 normal values were 0.14–0.54 g/l, ESR, normal values were 0–20 mm/hr; Prot, Yes indicates proteinuria >500 mg/day and nd indicates data was not available. Medications: Pred,10 mg/day prednisone; HCQ, hydroxychloroquine; MTX, methotrexate; AZP, azathioprine; MM, mycophenolate mofetil.

### Sample Acquisition, Phenotyping and Cell Sorting

Peripheral blood samples from study subjects were collected in heparinized tubes and immediately processed. PBMC were isolated using a brief density-gradient centrifugation over Ficoll-Hypaque [17]. Cells were kept at 4°C or on ice and cold buffers were employed to minimize alterations in gene expression during labeling and sorting. Cells were labeled with fluorochrome-conjugated antibodies obtained from BD Pharmingen (CD33-FITC, CD3-PE, CD4-PE-Cy5 and CD19-APC) and sorted using a 6-color (8-parameter) Cytomation MoFlo flow cytometer into individual subpopulations of CD19^+^CD3^−^ B cells, CD3^+^CD4^+^ T cells and CD33^+^CD3^−^ myeloid cells. Purity of the sorted populations was assessed by flow cytometry and was routinely ≥95%. Immunofluorescence labeling for flow cytometry was performed by incubating PBMC with anti-CD3-Cychrome, anti-CD4-APC, anti-CD8-PE, anti-CD19-PE, anti-CD38-APC and IgD-FITC antibodies in various combinations (all from BD Biosciences, San Jose, CA) for 30 minutes and washed as described [17]. Flow cytometry was performed using a FACSCalibur (BD Biosciences) with CellQuest software. Analysis was conducted with FloJo software [17].

### RNA Extraction and Chip Hybridization

RNA extraction, microarray analysis and quality control steps were performed according to validated protocols of the UT Southwestern Microarray Core (http://microarray.swmed.edu). Total cellular RNA was extracted from sorted cells using Trizol (GIBCO/BRL, Invitrogen, Carlsbad, CA) and RNA was further purified over RNeasy Qiagen columns (Qiagen Inc., Valencia, CA). RNA samples that were limited in amount or did not pass the quality control check on the bioanalyzer were excluded from further analysis. Purified biotin-labeled cRNA was generated and hybridized onto Affymetrix arrays, HG-U133A, using the Affymetrix protocol according to the manufacturer’s instructions. Each chip was scanned and subjected to Affymetrix algorithm analysis, normalized to the mean intensity of all values and data collected for further analysis [18].

### Protein Assays

Thioredoxin was measured by ELISA (Immuno-Biological Laboratories, Ltd., Japan) according to manufacturer’s instructions. The thioredoxin ELISA had a detection sensitivity of 3.9 ng/ml. Plasma thioredoxin levels were assessed by ELISA for an independent cohort of SLE patients (n = 8) and HC (n = 6) samples. Galectin-3 was measured by routine ELISA (Abnova Corporation, Walnut, CA) according to the manufacturer’s instructions. The galectin-3 ELISA had a detection sensitivity of 0.12 ng/ml. Plasma galectin-3 levels were determined for an independent cohort of SLE patients (n = 8) and HC (n = 8) samples.

### Microarray and Statistical Analysis

Genechip Operating Software v1.1 by Affymetrix was employed to generate gene expression values and the data was imported into GeneSpring v.7.3 (Silicon Genetics, Agilent Technologies, Palo Alto, CA) for further analysis following the manufacturer’s recommended protocols. Low intensity signals were filtered from the normalized data for the primary analysis by retaining only transcripts with a relative mean signal intensity of ≥200 on the array along with incorporation of the Cross-Gene Error Model. The fold change analysis and t-test were carried out on normalized filtered data. After applying the Cross Gene Error Model, lists of differentially expressed genes were obtained using the Welch’s t-test combined with the Benjamini Hochberg multiple testing correction to control for the false discovery rate (BH-FDR). The data was imported into Cluster for hierarchical cluster analysis and the results were visualized in TreeView (M. Eisen, Stanford, CA). The data is expressed as the ratio of the expression value to the mean expression value for each transcript and the ratios are depicted on a log_2_ scale in the dendrograms [12]. Red indicates transcripts expressed at higher levels relative to the mean, blue for transcripts expressed at lower levels than the mean, yellow for transcripts expressed at mean levels and black indicates no data. In order to maintain consistency in nomenclature for both cluster and pathway analysis, transcripts were annotated using italicized gene symbols. Information on particular genes was gathered from public databases, such as OMIM. In addition, networks were generated through the use of Ingenuity Pathways Analysis (IPA), Ingenuity® Systems. A 1.5 fold change cutoff was set to identify differentially regulated transcripts. Networks of differentially expressed genes were then algorithmically generated based on their product’s potential connectivity. A secondary analysis of differentially regulated transcripts was conducted with the normalized filtered data without a lower mean intensity signal threshold and using Welch’s t-test and GraphPad Prism v. 5.0 software (San Diego, CA) as has been described [19]. The normalized gene expression values were deposited into the Gene Expression Omnibus database at www.ncbi.nlm.nih.gov/geo (accession number GSE10325).Statistically significant differences were determined by unpaired t-tests for leukocyte cell counts, absolute numbers of cell subsets and phenotypic analyses by flow cytometry. The Mann Whitney U test was used to assess differences in flow cytometric assays and ELISA assays. The pearson correlation coefficient was used to assess the relationship between *CD38* signal intensity and SLEDAI scores.

## Results

### Peripheral Blood Leukocyte Subset Distribution in SLE

Leukocyte counts and differentials were carried out for blood samples studied in gene expression arrays. The absolute numbers of leukocyte subsets varied from patient to patient. In general, patients with active SLE (SLEDAI ≥8; N = 8) tended to have more severe leukopenia compared to those with inactive (SLEDAI ≤7; N = 6) disease (Figure S1). When the relative frequencies of the different leukocyte subsets were examined, we observed that neutrophils were elevated and lymphocytes were decreased in SLE patients as compared to healthy control samples (HC; N = 11). No statistically significant differences were observed in the total leukocyte counts or number of neutrophils, monocytes and basophils between HC and SLE patients with inactive or active disease.

### Gene Expression Profile of SLE B Cells

Three leukocyte subsets, CD19^+^CD3^−^ B cells, CD3^+^CD4^+^ T cells and CD33^+^CD3^−^ myeloid cells were sorted simultaneously from PBMC and assessed for gene expression by microarrays. The precision of our microarrays have been demonstrated previously [18]. We first compared a total of 14,478 transcripts from CD19+ B cells isolated from SLE patients (N = 14) to HC (N = 9). The analysis identified 193 probe sets recognizing 174 distinct transcripts with a ≥1.5 fold differential expression (p<0.05, Welch’s t-test, BH-FDR). A comprehensive list of the transcripts, fold change for inactive and active groups of SLE B cells compared to HC B cells and molecular functions are provided in the Table S1.

**Figure 1:**
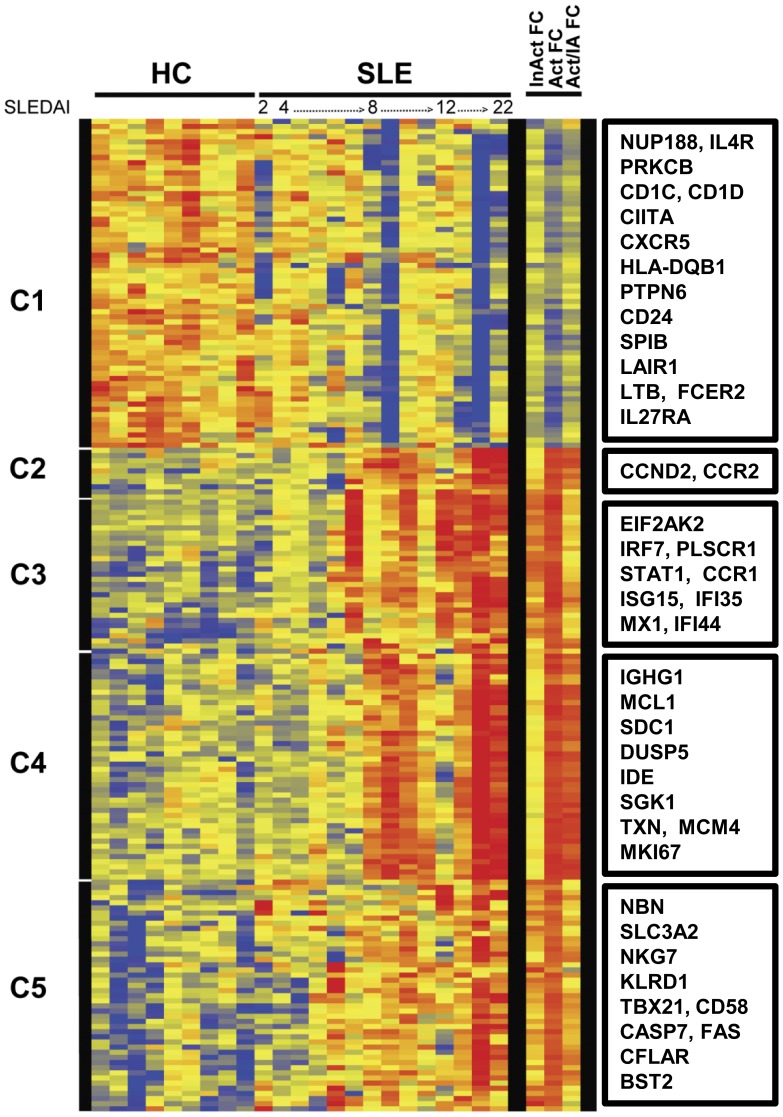
SLE B cell transcriptomes. Hierarchical cluster dendrogram of expressed genes that differed significantly between peripheral blood CD19+ B cells isolated from SLE patients and healthy controls (HC). The SLE samples are ordered by increasing SLEDAI score. The bars to the left indicate expressed gene clusters. Color changes indicate the expression level relative to the average (log_2_ scale). Red is increased expression, yellow is unchanged and blue is decreased expression. Indicated on the right are fold change in inactive SLE compared to HC (InAct FC), active SLE compared to HC (Act FC) and active SLE compared to inactive SLE (Act/IA FC). Select transcripts are identified for each cluster in boxes (right). For the entire list of transcripts in order as they appear on the dendrogram see supplementary Table S1.

Hierarchical cluster analysis was performed for the 174 transcripts differentially expressed in SLE B cells. The first four SLE patient samples (SLEDAI ≤4) clustered with the HC, whereas the SLE B cells from patients with active disease clustered together. The expressed gene clusters and samples ordered by SLEDAI scores were visualized as a heatmap (Figure 1). In order to maintain consistent nomenclature throughout both cluster and network analysis, gene symbols shown in italics, identify the transcripts. All gene symbols in the current study refer to the transcriptomes. Transcripts that were down-regulated in SLE B cells fell into a single cluster. Cluster 1 contained 64 probes which identified 55 down-regulated transcripts in SLE B cells. Of interest, transcripts for effectors of B cell signaling pathways including *FCER2* (CD23), *HLA-DQB1*, IL 4 receptor, alpha (*IL4R*) and the protein tyrosine phosphatase, non-receptor type 6 or SHP-1 molecule (*PTPN6*) were down-regulated in SLE B cells along with lymphotoxin-β (*LTB*), *CD24* and *CXCR5*.

The 119 transcripts identified by 129 probes that were up-regulated in SLE B cells fell into 4 clusters, with Clusters 3 and 5 encompassing expressed genes that were up-regulated in inactive and active SLE, while clusters 2 and 4 harbored transcripts that were up-regulated mostly in SLE patients with active disease, as delineated by the columns on the right in Figure 1. The 8 transcripts found in Cluster 2 were linked to diverse functions such as cell motility, C-C chemokine receptor 2 (*CCR2*) and growth regulation Cyclin D2 (*CCND2*). Type I IFN-inducible transcripts were enriched among the 26 expressed genes identified by 29 probes in Cluster 3. Transcripts of proteins responsive to extracellular signals, such as IL-6, TNF-α, IFN-γ or TGF-β, were in Cluster 4 which contained 44 transcripts identified by 47 probes and included the plasma cell marker, syndecan 1 (*SDC1*) or CD138, the serum/glucocorticoid-regulated kinase 1, SGK1 (*SGK1*), the dual specificity phosphatase 5, DUSP5 (*DUSP5*), Immunoglobulin Gm 1 (*IGHG1*) cell cycle control protein antigen KI-67 (*MKI67*) and the NFκB-regulated transcripts, *CANX* and *MCL1*. Finally, transcripts for apoptosis regulators (*CFLAR*, *CASP7*and *XAF1*), NK markers (*NKG7*, *KLRD1*), and IFN-inducible transcripts were enriched in Cluster 5 which was composed of 41 transcripts identified by 45 probes.

Many of the most highly expressed genes were well-known members of the IFN-inducible protein family, including IFI44 (10-fold up-regulated in active SLE) and it’s paralog, IFI44L (17-fold up-regulated in active SLE). Transcripts for *ISG15* and *RSAD2* were up-regulated by at least 9 fold in active SLE B cells as compared to HC. *CCR2, FNDC3B, GMNN, HERC5, IFI6, IFIT3, IFITM1, LAP3, MX1, NKG7, OAS3, PLSCR1, SPCS3, STAT1* were all up-regulated by at least 4-fold in active SLE B cells.

Network analysis of the transcripts that were differentially expressed in SLE B cells generated 7 networks populated with at least 15 differentially expressed transcripts. Examples of three of the most populated networks comprising several IFN-inducible molecules are shown in Figure S2.

### Gene Expression Profile of SLE T Cells

Similar to SLE B cells, the differentially expressed genes in SLE CD3^+^CD4^+^ T cells included a majority of up-regulated transcripts. When we compared a total of 14,402 transcripts from the CD4^+^ T cells of SLE patients (N = 14) to HC (N = 9), statistical analysis revealed 96 probe sets representing 92 unique transcripts that were differentially expressed at ≥1.5 fold threshold (p<0.05, Welch’s t-test, BH-FDR). Of these, 67 transcripts were up-regulated while 25 were down-regulated in SLE samples. The comprehensive list of the transcripts, fold change and molecular functions are provided in the Table S2.

**Figure 2:**
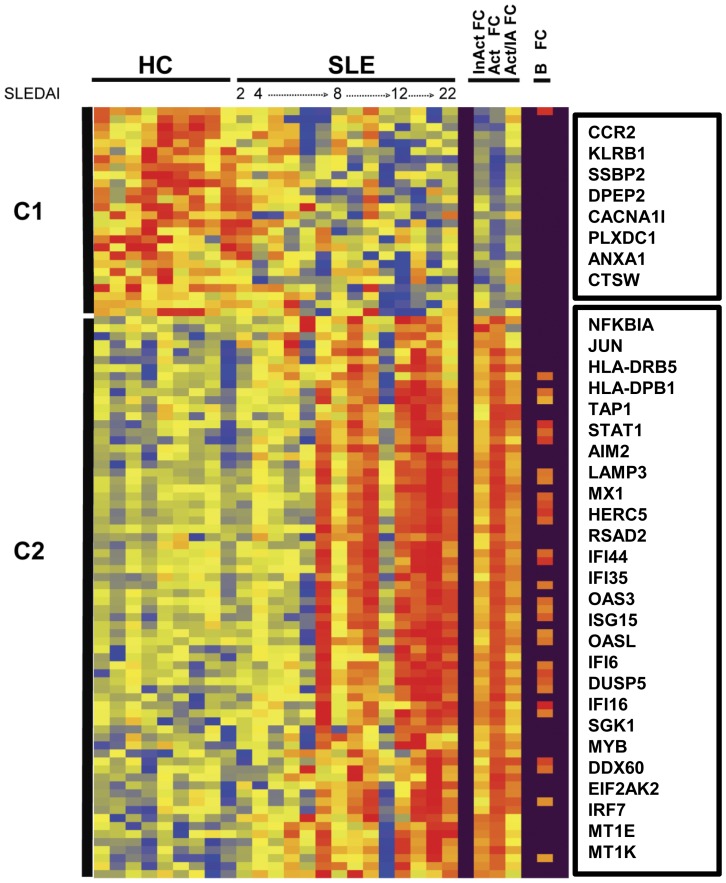
SLE CD4+ T cell transcriptomes. Hierarchical cluster dendrogram of expressed genes that differed significantly between sorted peripheral blood CD4+ T cells isolated from SLE patients and healthy controls (HC) as described in figure 1. Also indicated in the columns to the right are differentially expressed genes found in both B cell and T cell compartments of SLE (B FC). Select transcripts are identified for each cluster in boxes (right). For the entire list of transcripts ordered by clusters from the dendrogram see supplementary Table S2.

Hierarchical clustering of the expressed genes from CD4^+^ T cells revealed that the SLE patients with the highest SLEDAI scores over-expressed the majority of differentially regulated transcripts (Figure 2). Two clusters were observed in CD4^+^ T cells. Cluster 1 contained transcripts that were largely up-regulated in HC and down-regulated or not changed in the active SLE group including the membrane-associated protein tyrosine kinase activity (*EPHA1*) and the chemokine receptor, *CCR2*. Cluster 2 grouped up-regulated transcripts which were strongly expressed in active SLE T cells (Figure 2, right panel). A number of IFN-inducible transcripts were up-regulated in virtually all active SLE T cells (Table S2).

Among the differentially expressed genes, there were 29 transcripts which were shared between B cells and CD4^+^ T cells (Figure 2, far right panel); of these, 28 transcripts had a ≥2 fold change in active SLE CD4^+^ T cells and 12 transcripts had a ≥2 fold change in the inactive SLE CD4^+^ T cells. Almost all of the shared differentially expressed transcripts were coordinately up-regulated in both lymphocyte subsets, including *DUSP5* which negatively regulates the mitogen-activated protein kinase, ERK1 and the p38 stress kinase-induced kinase, serum/glucocorticoid regulated kinase I (*SGK1*). The kinase SGK1 can drive retinoic-acid-receptor-related orphan receptor γt (RORγt) and IL-23 receptor expression, as well as IL-17A and IL-17F production. An exception to the coordinate expression displayed by most transcripts was the chemokine receptor *CCR2*, which was up-regulated in SLE B cells but down-regulated in SLE CD4^+^ T cells. The most populated networks based on analysis of the CD4^+^ T cell transcripts is shown in Figure S3.

### Gene Expression Profile of SLE Myeloid Cells

**Figure 3:**
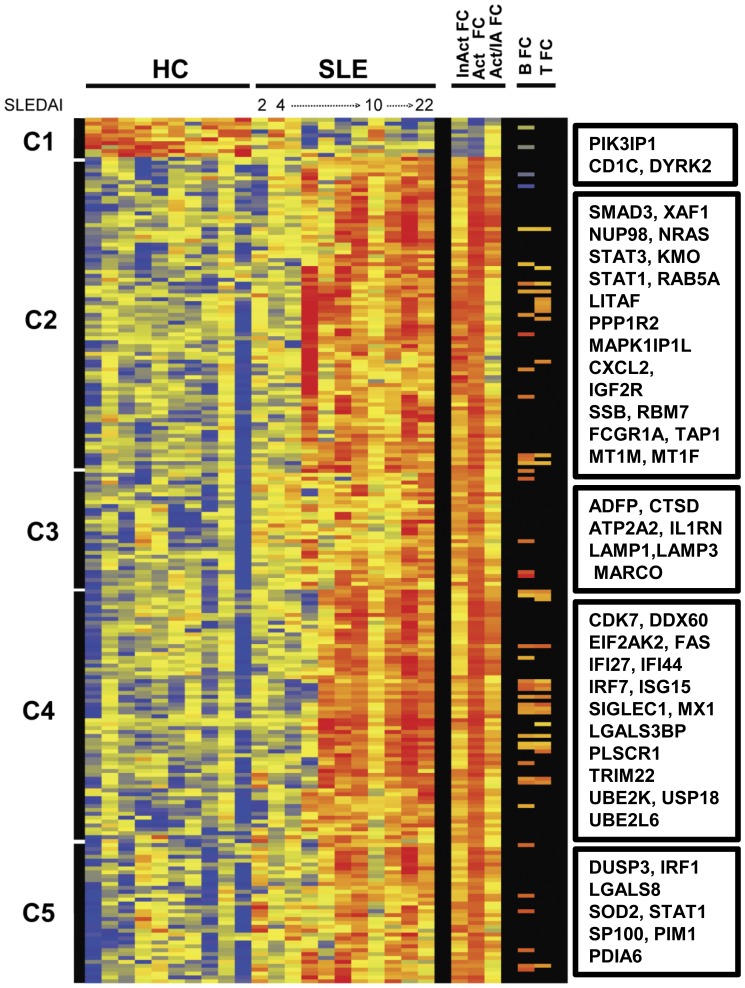
SLE Myeloid cell transcriptomes. Hierarchical cluster dendrogram of expressed genes that differed significantly between sorted peripheral blood CD33+ myeloid cells isolated from SLE patients and healthy controls (HC) as described in figure 1. Also indicated on the right are differentially expressed genes from the B cell compartment of SLE (B FC) or the T cell compartment of SLE (T FC) that were shared with myeloid cells. Select transcripts are identified for each cluster in boxes (right). For the entire list of transcripts ordered by clusters from the dendrogram see supplementary Table S3.

Of the 14,652 transcripts present in CD33^+^CD3^−^ myeloid cells, we identified 222 probe sets for 208 unique transcripts with a ≥1.5 fold differential expression (p<0.05, Welch’s t-test, BH-FDR) from SLE patients (n = 11) compared to HC (n = 10). There were 198 up-regulated transcripts and 10 down-regulated transcripts. The comprehensive list of the gene names, fold changes and molecular functions are provided in the Table S3. Hierarchical clustering of the expressed genes from CD33^+^CD3^−^ myeloid cells generated 5 clusters (Figure 3). The 10 down-regulated transcripts observed in Cluster 1 were found in both inactive and active SLE myeloid cell groups. Included in this cluster were transcripts for a cell surface molecule *CD1C*, the ribosomal proteins L18a and L22 (*RPL18A* and *RPL22*) and a dual specificity tyrosine phosphorylation-regulated kinase 2 transcript (*DYRK2*). DYRK2 is a serine/threonine-protein kinase involved in the regulation of cell growth and differentiation.

The analysis revealed that myeloid cells from the inactive SLE group expressed up-regulated transcripts primarily in cluster 3 with fewer up-regulated transcripts in the other clusters, whereas active SLE myeloid cells exhibited up-regulated transcripts in clusters 2 through 5 (Figure 3, right panel). Cluster 2 contained 81 probe sets for 74 transcripts including transcripts such as *CXCL2, FCGR1A, STAT1* and *STAT3*. Cluster 3 was composed of 28 probe sets for 27 up-regulated transcripts including one for the interleukin-1 receptor antagonist (*IL1RN*) and the cell surface receptor *MARCO*, which can serve as a scavenger receptor. Transcripts for two histone proteins (*HIF0* and *H1FX*), the lysosome and endosome-associated molecules, CD107a (*LAMP1*) and *CD63* as well as other molecules such as adipocyte differentiation-related protein, *ADFP* were also up-regulated in SLE myeloid cells. Cluster 4, with 68 probe sets for 62 transcripts included CD169 (*SIGLEC1*), *LGALS3BP*, IFN-inducible *IRF7, ISG15, MX1* and cell cycle regulators (*CDK7* and *CALM3*). The last cluster of 35 probe sets to unique transcripts, Cluster 5, encompassed a kinase, *PIM1*, a phosphatase (*DUSP3*), IFN-inducible transcripts and various enzymes including *PDIA6* and *SOD2*. Examples of two of the most populated networks generated by analysis of myeloid cell transcripts are shown in Figure S4.

**Figure 4:**
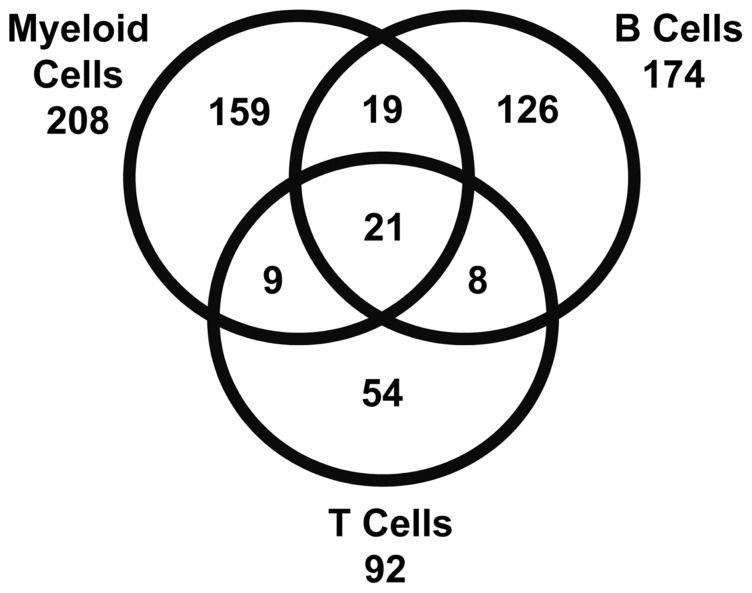
SLE Subsets Up-regulate Unique Transcriptional Profiles. A Venn diagram demonstrating shared and unique differentially expressed transcripts of SLE myeloid cells, B cells and T cells. Of the 474 combined transcripts only 4.4% (21) were shared by all three subsets, whereas 69% (329) of the transcripts were unique to a particular subset at the threshold set for the described primary analysis.

Forty transcripts were shared between myeloid cells and B cells (Figure 3, right). SLE myeloid cells and CD4^+^ T cells shared 30 of the differentially expressed genes (Figure 3, far right). Of these, 21 transcripts were differentially expressed in all three peripheral blood subsets from SLE patients (Figure 4, Table 2). The shared transcripts were mostly well-known IFN-inducible transcripts including *IRF7, MX1* and *STAT1*. As shown in the Venn diagram (Figure 4) a majority of highly expressed SLE transcripts were unique for either B cell or myeloid cell subsets, whereas approximately half of the CD4^+^ T cell transcripts were shared with the other subsets. Of the shared expressed genes, only one down-regulated transcript, *CD1C*, was shared between B cells and myeloid cells, while *LAIR1* was down-regulated in B cells and up-regulated in myeloid cells. Other than *CCR2* in CD4^+^ T cells as discussed above, the remainder of shared transcripts were up-regulated.

**Table 2:** Transcripts shared by SLE subsets.

Gene Symbol	Entrez Gene Name	Location	Type
DDX60	DEAD (Asp-Glu-Ala-Asp) box polypeptide 60	Unknown	other
EIF2AK2	eukaryotic translation initiation factor 2-alpha kinase 2	Cytoplasm	kinase
HERC5	hect domain and RLD 5	Cytoplasm	enzyme
IFI6	interferon, alpha-inducible protein 6	Cytoplasm	other
IFI35	interferon-induced protein 35	Nucleus	other
IFI44	interferon-induced protein 44	Cytoplasm	other
IFI44L	interferon-induced protein 44-like	Unknown	other
IFIT3	interferon-induced protein with tetratricopeptide repeats 3	Cytoplasm	other
IRF7	interferon regulatory factor 7	Nucleus	transcription regulator
ISG15	ISG15 ubiquitin-like modifier	Extracellular Space	other
LAP3	leucine aminopeptidase 3	Cytoplasm	peptidase
MT1X	metallothionein 1X	Unknown	other
MX1	myxovirus (influenza virus) resistance 1	Nucleus	enzyme
N4BP1	NEDD4 binding protein 1	Cytoplasm	other
OAS3	2′-5′-oligoadenylate synthetase 3, 100 kDa	Cytoplasm	enzyme
OASL	2′-5′-oligoadenylate synthetase-like	Unknown	enzyme
PLSCR1	phospholipid scramblase 1	Plasma Membrane	enzyme
RSAD2	radical S-adenosyl methionine domain containing 2	Unknown	enzyme
STAT1	signal transducer and activator of transcription 1, 91 kDa	Nucleus	transcription regulator
TAP1	transporter 1, ATP-binding cassette, sub-family B (MDR/TAP)	Cytoplasm	transporter
UBE2L6	ubiquitin-conjugating enzyme E2L 6	Cytoplasm	enzyme

Transcripts up-regulated in SLE CD19^+^ B cells, SLE CD3^+^ CD4^+^ T cells and SLE CD33^+^ myeloid cells as compared to the same subsets isolated from healthy subjects. For details see the supplementary tables.

As has been reported by others, we confirmed elevation of expression of transcripts, such as 2′5′-oligoadenylate synthetase 1 (*OAS1*) and ISG12 also known as IFI27 (*IFI27*), by Taqman RT-PCR and normalization of sample cycle threshold to *GAPDH* in a second cohort of SLE patients as compared to healthy subjects (data not shown) [20], [21].

### Gene Expression Profiles Regulated by IFNs

**Figure 5:**
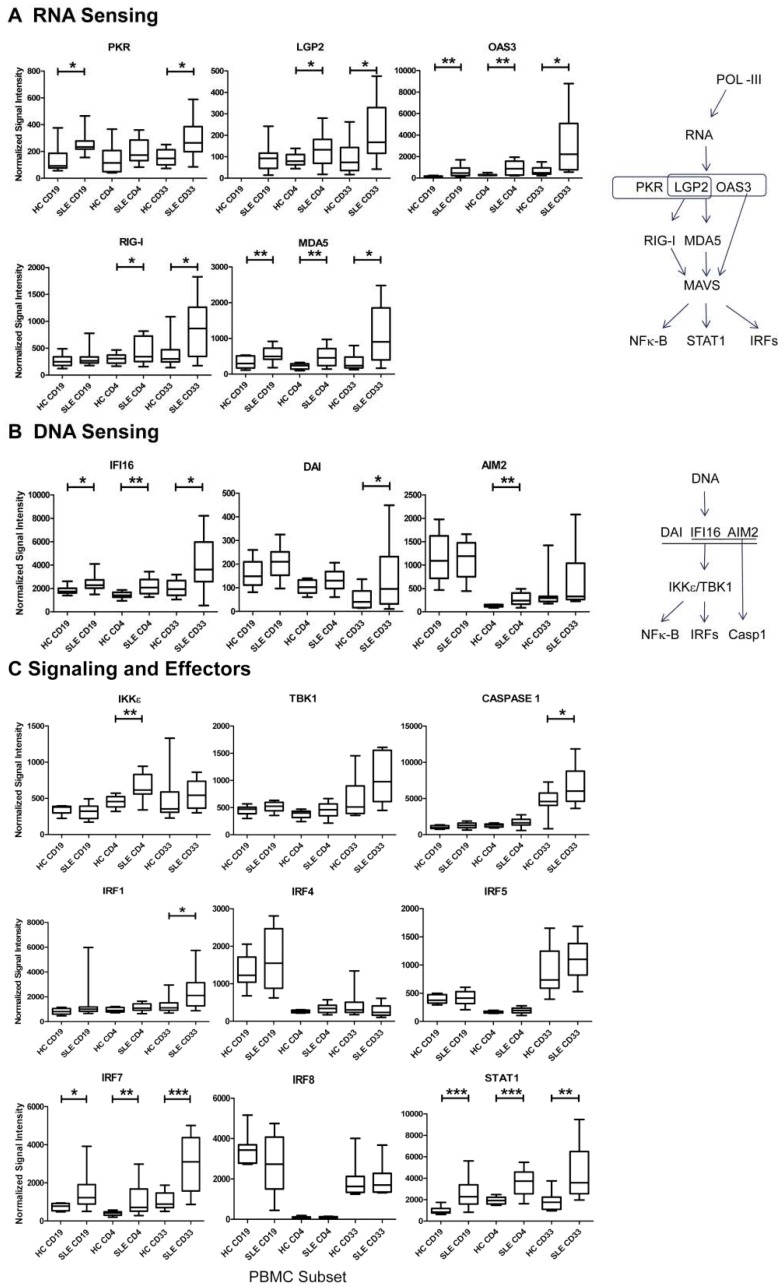
DNA and RNA Sensors are Transcriptionally Active in SLE Subsets. A secondary analysis was performed with all normalized data in order to compare transcripts for specific pathways (19). (A) RNA sensing molecules (B) DNA sensing molecules and (C) downstream signaling and effector molecules were up-regulated in the three subsets as shown (Welch’s t-test, p<0.05*, p<0.01**, p<0.001***).

Although the data suggested transcriptional activation of a number of IFN-regulated networks in SLE PBMC, few of the listed transcripts were expressed in canonical pathways assessed by a variety of available tools. Therefore, we interrogated IFN-regulated transcript differences between the SLE and HC samples by a second method as reported [19]. For these studies we first assessed IFN-regulated pathway members that were described by Li et al. to be transcriptionally up-regulated as determined by real-time PCR in murine innate cells in response to IFN-β or a viral stimulus [22]. In response to IFN-β, Li et al. observed up-regulation for transcripts of five cytosolic RNA sensing molecules PKR, LGP2, RIG-I, MDA5 and OAS3. As noted in Table 2, OAS3 and the RNA-dependent protein kinase R, PKR (*EIF2AK2*) were significantly up-regulated in all three SLE PBMC subsets. As observed in Figure 5A, PKR and LGP2 were also up-regulated in all SLE PBMC subsets. In addition to being a cytosolic nucleic acid sensor, LGP2 which is an RNA helicase encoded by *DHX58*, is a positive regulator of MDA5 (*IFIH1*) and RIG-I (*DDX58*) [23]. As can be seen, the transcripts for MDA5 (*IFIH1*) and RIG-I (*DDX58*) were also up-regulated in SLE subsets as compared to HC. Likewise, we noted increased gene expression of the DNA sensing pathway members IFI16 (*IFI16*), DAI (*ZBP1*) and AIM2 (*AIM2*) in SLE subsets as shown in Figure 5B. No transcriptional upregulation of endosomal nucleic acid sensing TLRs was noted by Li et al. after murine innate immune cell stimulation, however, we did observe a modest increase in *TLR7* B cell gene expression (data not shown). Transcripts for distal pathway members, such as *MAVS*, were not differentially regulated in the studies by Li et al. or in our analysis. We observed a trend toward increased gene expression in certain subsets for downstream pathway members IKKε (*IKBKE*), TBK1 (*TBK1*) and significant increases in the expression of multiple transcription factors as mentioned above, such as *IRF1* and *STAT3* in myeloid cells and *IRF7* and *STAT1* in all SLE PBMC subsets, as shown in Figure 5C. *IRF3* was expressed at a similar level in all subsets from HC and SLE patients. Also, little change in *IRF5* transcriptional activity could be discerned between HC and SLE subsets although increased expression was observed in the myeloid subsets (Figure 5C). *IRF4* was highly expressed by B cells and there was increased expression in active SLE B cells as compared to inactive SLE B cells (not shown). By contrast, *IRF8* had similar transcriptional activity in inactive SLE B cells compared to HC, whereas active SLE B cell *IRF8* gene expression was decreased by 40% (not shown). *IRF8* expression levels in T cells and myeloid cells were not different between subsets from HC and SLE patients (Figure 5C).

### Elevated Protein Expression in SLE Blood

**Figure 6:**
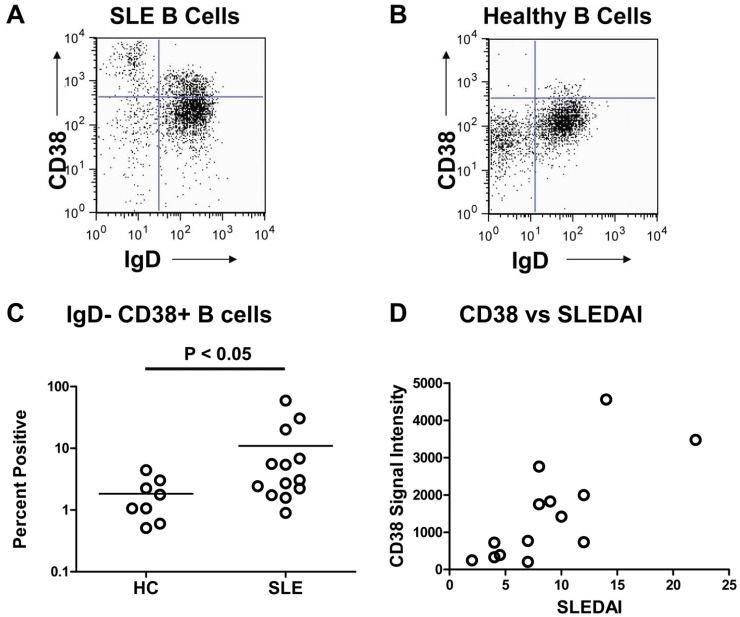
Validation of Array Results by CD38 Protein Expression. (A) Increased CD38^+^ IgD^−^ expression by CD19^+^ gated B cells from an SLE patient and (B) CD38^+^ IgD^−^ expression by CD19^+^ gated B cells from a HC. A total of 5×10^4^ PBMC were assessed from blood studied in the gene expression arrays by incubation with anti-CD38-APC, anti-IgD-FITC and anti-CD19-PE, followed by flow cytometric analysis. (C) Frequency of CD38^+^ IgD^−^ CD19^+^ B cells was assessed on available PBMC analyzed for gene expression. There was a statistically significant difference (P<0.05) in CD38^+^ IgD^+^ CD19^+^ B cells between SLE patients (n = 13) and healthy controls (n = 8). (D) *CD38* signal intensity values for transcripts obtained from microarrays correlated (r_P_ = 0.7542 and *P*≤0.005) with SLEDAI scores for SLE B cells (n = 14).

We initiated studies to examine the protein levels of differentially expressed transcripts. Several molecules were assessed by flow cytometry for comparison with transcripts from the PBMC subsets. SLE B cells demonstrated up-regulated expression of *CD38* transcripts by 3-fold compared to HC in the arrays and there was a significant difference between means as assessed using a secondary analysis (p<0.05, Welch’s t-test). B cells from aliquots of PBMC employed in the microarrays were examined for CD38 protein expression by flow cytometric analysis (Figure 6A–C). As expected, we found elevated CD38 cell surface expression on SLE B cells as compared to HC. CD38 is known to be a marker for both activated B cells and plasmablasts, both of which are elevated in the periphery of SLE patients. We examined the correlation between *CD38* transcriptional activity and disease activity. As shown in Figure 6D, increased expression of *CD38* transcripts correlated with active disease (r_P_ = 0.7542 and p ≤ 0.005). Thus, the microarray results for *CD38* were in agreement with the flow cytometric analysis for SLE B cells from the same patient samples.

**Figure 7:**
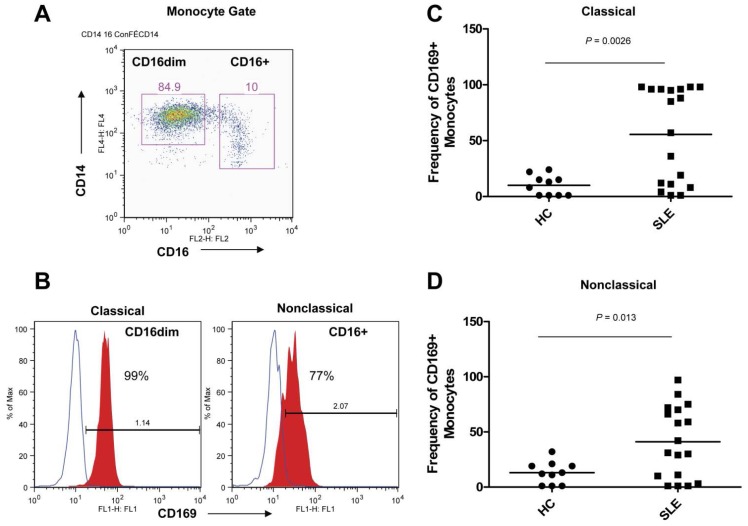
Confirmation of Myeloid Array Results by CD169 Protein Expression . The high expression of CD169 (*SIGLEC1*) by myeloid cells in the arrays was confirmed by analysis of CD33^+^CD14^+^ myeloid cells in a second cohort. (A) CD14^+^ myeloid cells were gated for CD16^dim^ and CD16^bright^ myeloid cells. (B) Histograms of CD169 expression on classical (CD14^+^CD16^dim^) and nonclassical (CD14^+^CD16^+^) myeloid cells. (C) Frequency of CD169^+^ cells in the classical (CD14^+^CD16^dim^) myeloid subset comparing HC to SLE patients. (D) Frequency of CD169^+^ cells in the nonclassical (CD14^+^CD16^+^) myeloid subset comparing HC to SLE patients.

CD169 (*SIGLEC1*) was the most highly expressed cell surface molecule in myeloid cells. CD169 was up-regulated by more than 4-fold in inactive SLE and 11-fold in active SLE myeloid subsets. The high expression of CD169 by myeloid cells in the arrays was confirmed by analysis of CD33^+^CD14^+^ myeloid cells in a second cohort of healthy and SLE samples (Figure 7). Increased protein expression of CD169 was observed on both classical (CD14^+^CD16^dim^) and nonclassical (CD14^+^CD16^+^) myeloid cells, the later subpopulation was found in increased numbers in active SLE PBMC. We also examined the intracellular expression of the lysosome and endosome-associated molecules, CD107a (*LAMP1*) and CD63 (*CD63*) in a second cohort of HC and SLE samples. An increased mean fluorescence intensity (MFI) in CD63 protein expression was easily detected in SLE CD4^+^ T cells and CD33^+^CD14^+^ myeloid cells and these subsets were virtually all positive for CD63 by flow cytometry. CD107a protein, which was selectively expressed in myeloid cells, was also elevated in the SLE myeloid subset compared to matched HC as shown in Figure S5.

The antioxidant, thioredoxin (*TXN*), was found to be transcriptionally up-regulated in both SLE B cells and SLE myeloid cells. In validation studies using an orthogonal approach, thioredoxin protein was confirmed to be up-regulated in plasma samples from SLE patients compared to HC (Figure S6A). Preliminary results indicated that galectin-3 was also significantly increased in the plasma of SLE patients (Figure S6B). These results suggested that some of the genes up-regulated in cells at the transcriptional level, in both the periphery and likely more so by cells at the inflammatory site, were more abundant in SLE serum at the protein level. This observation has important implications for biomarker discovery.

## Discussion

This work revealed distinct transcriptional profiles in SLE CD19^+^ B cell, CD4^+^ T cell and CD33^+^ myeloid cell subsets isolated from peripheral blood. Although a number of SLE susceptibility genes have been reported, recent evidence has highlighted the potential relevance of gene expression studies. For example, Furukawa et al. found no differences in genetics with regard to single nucleotide polymorphisms or copy number variations between monozygotic twins discordant for SLE, however variations were observed in gene expression and epigenomic analyses [24]. Our results confirm that a number of IFN-inducible transcripts are elevated in all three SLE PBMC subsets, and their expression correlated with disease activity. Elevated expression of a number of transcripts related to cytokine activation, cell differentiation, cell cycle regulation, and apoptosis were also observed in SLE PBMC subsets suggestive of increased activation and differentiation as compared to cells from healthy subjects [17], [25], [26].

We had previously noted that patients with the most active disease had the most variable leukocyte counts [17]. The variability observed in SLE patients is consistent with the leukopenia and lymphopenia reported in the literature [27]–[29]. Previous array studies have reported significant differences between SLE PBMC and healthy subjects [12]. Given the fact that the cell composition of the peripheral blood in SLE patients differs from that of healthy individuals, previous microarray studies performed using whole blood might be interpreted with caution. The current study more accurately reflects cell-type specific differences in SLE transcripts.

Our studies reproduced the IFN signature found in whole blood. In a recent report, 21 IFN-inducible transcripts were selected as candidate pharmacodynamic markers from arrays of SLE whole blood, validated by TaqMan qRT-PCR and further confirmed with microarrays on a second cohort [20]. Of the 21 up-regulated transcripts, 19 were confirmed in our SLE subsets. Ten were expressed by all 3 cell subsets (*HERC5*, *IFI6*, *IFI44*, *IFI44L*, *IFIT3*, *ISG15*, *MX1*, *OAS3*, *PLSCR1* and *RSAD2*). Four transcripts (*IFI27*, *IFIT1*, *SIGLEC1* and *USP18*) were hyper-expressed exclusively in myeloid cells, two were elevated in both T and myeloid cells (*LOC26010* also known as *SPATS2L* or *DNAPTP6* and *CD63*), two were increased in both T and B cells (*OAS2* and *RTP4*), and one was increased exclusively in T cells (*OAS1*). Two transcripts were not found in our analysis, epithelial stromal interaction 1 (*EPSTI1*) and lymphocyte antigen 6 complex, locus E (*LY6E*). There was no probe set for *EPSTI1* on our arrays. *LY6E* was elevated in SLE compared to HC as it was 2-fold up in SLE B and SLE T cells and 5-fold increased in SLE myeloid cells (p = 0.003 by Welch’s t-test), however, it was excluded in our primary analysis for overall variability in signal intensity. Although a number of up-regulated SLE transcripts were found in earlier arrays, few transcripts were shared between multiple studies as a result of varying technologies [12]–[15]. However, there was an excellent correlation between the up-regulation of gene expression in SLE whole blood and PBMC as reported by Yao et al. with the expression profiles in purified SLE B-, T- or myeloid cells in our study [20].

Several differentially expressed SLE transcripts have been implicated as having a potentially significant role in disease. An autoimmune disease risk variant for MDA5 (*IFIH1*), which binds dsRNA and results in increased sensitivity to IFN-α has been described [30]. Likewise, over-expression of MDA5 in a murine model resulted in viral resistance and when combined with a lupus-susceptible background, acceleration of autoimmune nephritis [31]. Single nucleotide polymorphisms in the genes for *IRF5*, *IRF7* and *IRF8* have also been associated with an increased risk for SLE and other autoimmune diseases [32]. While variants of *IRF5* are thought to contribute to enhanced IFN-α production, little is known about the role of *IRF7* and *IRF8* in SLE [33], However, *IRF4* and *IRF8* are known regulators of B cell differentiation [34], [35]. Despite the many up-regulated IFN-inducible transcripts, a number of transcripts key to the induction of IFN or nucleic acid sensing pathways were not up-regulated in our studies or in the work by Li et al. [22]. Therefore, the similarities in increased transcripts for cytosolic RNA versus DNA sensors was notable. Whereas, the RNA sensors RIG-I, MDA5, OAS3, LGP2 and PKR were elevated in both studies, the DNA sensors IFI16 and DAI were transcriptionally active in particular subsets only in our study. AIM2 (*AIM2*) was not examined in the Li et al. study. Recent work has established that AIM2 is the only dsDNA sensor that is found exclusively in the cytoplasm. AIM2 can also form a heterodimer with IFI16, which is found primarily in the nucleus. Both of these molecules belong to the PYHIN family which includes murine *IFi202a* encoding the p202a protein. Of interest, *IFi202a* is a candidate lupus susceptibility gene and p202a acts to negatively regulate the AIM2 inflammasome and several transcription factors including NF-κB and AP-1 [11], [36]–[38]. Recent studies clearly establish a link between AIM2 binding dsDNA and activation of the inflammasome, as reviewed by Calvar et al. [36]. More studies in this area are warranted regarding the role of cytosolic RNA and DNA sensors in lupus, hyper-expression of IFN-α and the subsequent impact on effector functions [39].

SLE B cells possessed approximately 30% more differentially expressed transcripts than their CD4+ T lymphocyte counterparts. Our network analysis suggested that STAT3 regulated pathways were activated in SLE B cells. STAT3 can be activated by IL-6, IL-10 and IL-21, of which both IL-6 and IL-10 were elevated in serum samples of our SLE cohort (data not shown). All 3 cytokines were reported to be elevated in murine lupus and human SLE [40]–[43]. Moreover, IL-6 potentiates the effects of type I IFNs in STAT1 activation and the activation of SGK1, a stress kinase that was transcriptionally up-regulated in both T and B cells. The MAPK pathway was also highlighted in network analysis. Nicholas et al. [44] demonstrated that CD19^hi^ B cells had increased basal levels of phosphorylated Syk and ERK1,2. Similarly, B prosurvival signaling required sustained activation of Akt and ERK kinases which were associated with elevated anti-apoptotic proteins Mcl-1, Bcl-xL and XIAP [45]. We reported similar signaling changes in murine lupus [46]. The network analysis results support previous studies indicating a role for both cytokines and autoantigen induced BCR activation of signaling pathways in peripheral SLE B cells.

The transcriptional profiles in SLE B cells are likely to have been influenced by altered subset composition in lupus, relative to HC. Indeed, previous reports have noted an increase in the number of early B cells, memory B cells and CD38^+^ plasmablasts in lupus in both murine models and patient samples [25], [26], [46]–[50]. Several transcripts connected with germinal center activity were down-regulated in SLE B cells. These included transcripts for CXCR5 (*CXCR5*), which is required for the follicular localization of B cells, LT-β (*LTB*) which is required for germinal center organization, and Spi-B (*SPIB*) which is required for the maintenance of germinal centers [51]–[53]. Other surface markers down-regulated with B cell differentiation (e.g., to plasmablasts) included transcripts for Class II molecules and *FCER2* (CD23). In contrast, *CCR1* and *CCR2* transcripts were up-regulated in SLE B cells. These markers are not commonly found in germinal centers, but could be associated with more differentiated B cells. The increased levels of syndecan-1 or CD138 (*SDC1*), *IRF4* and *Ig* transcripts in SLE B cells is also consistent with increased B cell differentiation. *CD58* (LFA-3) was notably up-regulated in SLE B cells from both patients with inactive and active disease. CD58 protein, which binds to CD2 on T cells, is an important adhesion molecule between T cells and antigen presenting cells (APC). Thus, the detection of increased protein expression of CD58 on peripheral B cells suggests their potential as APC. NK cell related transcripts were also up-regulated in SLE B cells with active disease. Of note, early B cells have been documented to express transcripts for T and NK markers which were down-regulated with maturation [54]. Finally, in a second analysis we found additional patterns of gene expression consistent with SLE B cell activation and differentiation to plasmablasts, e.g., increased *CD38*, *XBP-1* and decreased *CIITA*, which have been shown to correlate with increased disease activity scores. T-bet (*Tbx21*) expression was increased in both inactive and active SLE B cells. T-bet is important for class-switch recombination and type I IFN can up-regulate T-bet expression [11], [55], [56]. Consistent with these observations, a deficiency in a single transcription factor, Ets-1, has been shown to drive an activated B cell phenotype and increased differentiation to plasma cells which resulted in autoimmune disease [57]. Finally, Wu et al. described the activation of several cell cycle related molecules in murine lupus B cells [46]. Cell cycle-related transcripts were elevated in SLE B cells in our study including cyclin D2 (*CCND2*) and Ki67 (*MKI67*). Thus, these findings suggest the presence of actively cycling cells in the periphery of SLE patients which may relate to the lack of retention of cells, such as plasmablasts, in secondary lymphoid organs as a result of altered expression of cytokines and chemokines and their receptors as described [39].

Several interesting transcripts were up-regulated in SLE T cells. A number of metallothioneins were up-regulated in SLE T cells suggesting active regulation of the redox state which could result from cell activation and/or chemotaxis [58], [59]. The up-regulated transcripts for c-Jun (*JUN*) and SGK1 were also observed. Both are targets of the p38 MAPK stress kinase pathway and c-Jun is a target of the antigen-driven Ras/MAPK pathway (Figure S3). c-Jun has been shown to interact metallothioneins (*MT1F* and *MT2A*) in addition to *MYB* [60]. SGK1 has recently been shown to be a key regulator of pathogenic autoreactive Th17 cells [61], [62]. Our findings support previous studies describing the aberrant activation state of peripheral blood SLE T cells [63]–[66]. We noted the robust expression of certain IFN-inducible transcripts in response to IFN-α stimulation [67]. *DUSP5* expression was exclusively induced by IL-12. IL-12 was only detectable in the serum of 3 SLE patients (data not shown). These findings suggest that serum cytokine levels do not reflect systemic cytokine levels. Thus, cells activated at inflammatory sites could be present in the peripheral circulation. Increased *CREM* transcripts encoding for CREM2 were observed in both inactive and active SLE CD4^+^ T cells. Increased CREM2 protein expression has been linked to an IL-2 deficiency in SLE T cells, mediated by CREM-2 transcriptional repression on the FOS promoter which resulted in insufficient c-Fos expression [68].

The broad SLE myeloid transcriptome included transcripts for molecules that fell into a number of different pathways. *CXCL2*, *FCGR1A* (CD64), *SIGLEC1* (CD169), *STAT1* and *STAT3* transcripts, all known to be associated with inflammatory responses, were up-regulated in SLE myeloid cells. Moreover, p21^(WAF1/CIP1)^, the stress responsive cyclin-dependent kinase inhibitor (*CDKN1A*) was increased in SLE myeloid cells. p21^(WAF1/CIP1)^ prevents DNA replication and has been associated with regulation of inflammatory cytokines [69]. Moreover, an SLE-associated SNP at position −899 in the promoter of the minor allele A of *CDKN1A* irrespective of *HLA-DRB1* alleles has been reported [70]. *ETV6* (ETS translocation-variant gene 6) and *ETV7*, both up-regulated in SLE myeloid cells, function as transcriptional repressors and their expression was consistent with cell activation [71], [72]. The elevated expression of these and other transcripts is in agreement with the activated state of myeloid cells in the SLE.

Our studies demonstrate that protein expression correlated with gene expression. *CD38* (ADP ribosyl cyclase) transcripts were increased in B cells from active SLE patients and we observed elevated cell surface CD38 expression on active SLE IgD^−^ B cells from the same patients. Increased numbers of CD38 expressing pre-naïve and naïve B cells have also recently been found in the blood of active SLE patients [73]. Transcripts for CD169 (*SIGLEC1*) were up-regulated in SLE myeloid cells. Recent studies indicate that CD169 expression is increased on SLE inflammatory monocytes [74].

We asked if elevated transcripts in SLE leukocytes translated to elevated proteins in the serum or plasma. To this end, we assessed serum and plasma samples for various proteins that might also serve as surrogate biomarkers. One such candidate that showed promise was thioredoxin (*TXN*), a ubiquitous antioxidant with anti-apoptotic properties. This molecule was elevated in SLE plasma, consistent with the transcriptional profile observed in B and myeloid cells. Systemic expression of thioredoxin could potentially impact peripheral cell activation, proliferation and differentiation in SLE. Another molecule, galectin-3 (*LGALS3*), which was transcriptionally up-regulated in SLE B cells, was also elevated in SLE plasma. Galectin-3 and galectin-3 binding protein, LGALS3BP were elevated in rheumatoid synovial fluid, while galectin-3 was elevated in sera of rheumatoid arthritis patients [75]. The galectin family members are thought to contribute to inflammation and tumor metastasis [76], [77]. *LGALS3BP* expression was elevated in both T cells and myeloid cells. Collectively, these preliminary leads raise the possibility that other elevated transcripts in SLE leukocytes might be indicative of increased SLE plasma protein expression. These findings have important implications for the design of multiplex biomarker assays for monitoring SLE.

In sum, the current studies confirm the presence of an IFN signature in peripheral B cells, T cells and myeloid cells isolated from SLE patients with active disease. A majority of transcripts up-regulated in each SLE subset was unique to the particular cell type. Thus, B cells and myeloid cells expressed transcripts associated with cell-type specific activation and differentiation to a proinflammatory phenotype [39], whereas T cells expressed a profile consistent with enhanced transcriptional regulation. Transcripts for cytosolic RNA sensors, DNA sensors and their downstream targets in pathways mediating cytokine production were most highly expressed in myeloid cells and up-regulated in all subsets in concert with increased disease activity. These studies support a role for the upregulation of cytosolic nucleic acid sensing pathways as a mechanism driving inflammation involving both lymphocytes and myeloid cells. Prolonged activation of these pathways could result in sustained cytokine production which might incite inflammation and destruction of target tissues.

## Supporting Information

Figure S1 **Leukocyte subset distribution in peripheral blood.** (Upper panel) Cell counts and differentials were performed on blood samples collected for microarrays. Leukocyte, lymphocyte and monocyte counts are shown for healthy controls (HC) and inactive (SLEDAI ≤7) or active (SLEDAI ≥ 8) SLE samples. (Lower panel) Absolute numbers of lymphocytes were calculated from the cell count multiplied by the frequency of each subset. The frequency of HC and SLE lymphocytes were: CD3^+^CD4^+^ T cells (HC, 42% ±9%; median ± SD, N = 8 versus SLE, 30% ±12%; N = 13); CD3^+^CD8^+^ T cells (HC 19% ±5%, N = 8 versus SLE 20% ±7%, N = 12); CD3^−^CD19^+^ B cells (HC, 12% ±3%, N = 8 versus SLE 7% ±5%, N = 13). Frequencies of neutrophils, lymphocytes and monocytes in HC versus SLE were: neutrophils (54.7% ±9.9 vs 63.5% ±13.6; mean ± SD), lymphocytes (35.1% ±9.4 vs 26. 9±11.3) and monocytes (6.0% ±1.7 vs 7.1±1.5). Frequencies for SLE groups with inactive versus active disease were: neutrophils (57.6% ±15.4 vs 67.9% ±10.9), lymphocytes (31.6% ±13.1 vs 23.4±9.0) and monocytes (7.7% ±1.1 vs 6.7±1.6). These relative subset differences attained statistical significance when active SLE was compared to HC for neutrophils (p<0.05) and lymphocytes (p<0.05). Absolute numbers of SLE lymphocytes were decreased in inactive (p<0.05) and active disease (p<0.005) compared to HC. Absolute numbers of CD19^+^CD3^−^ B cells and CD3^+^CD4^+^ T cells were significantly decreased in SLE patients (lower panel) and CD3^+^CD8^+^ T cell numbers were lower in patients with active disease. There was an increased frequency of SLE double negative lymphocytes (40.9% ±19.32, N = 12) as compared HC (28.27% ±12.95, N = 8). The greatest differences were observed in the relative frequencies of neutrophils and monocytes versus lymphocytes.
(TIF)

Figure S2 **Network analysis of differentially expressed transcripts in SLE B cells.** Differentially expressed transcripts in SLE B cells were tested for inter-relationships based on the published literature using IPA to obtain networks (A–C).The up-regulated expressed genes, relative to HC B cells are red with increasing intensity corresponding to increasing fold change and down-regulated expressed genes are shown in green. Direct interactions (binding or direct regulation) between products of transcripts are shown with solid lines and indirect relationships are shown using interrupted lines, as deduced from the published literature.
(TIF)

Figure S3 **Network analysis of differentially expressed transcripts in SLE CD4+ T cells.** Differentially expressed transcripts in SLE CD4+ T cells were tested for inter-relationships based on the published literature, using IPA as described in Figure S2.
(TIF)

Figure S4 **Network analysis of differentially expressed genes in SLE myeloid cells.** Differentially expressed transcripts in SLE myeloid cells were tested for inter-relationships based on the published literature. Networks (A and B) assessed with IPA as described in Figure S2.
(TIF)

Figure S5 **Elevated Endosomal Proteins in SLE T Cells and Myeloid Cells.** Anti-CD3 and anti-CD4 antibodies were employed to identify CD4+ T cells. Anti-CD14 and/or anti-CD33 antibodies were utilized to identify monocytes. (A,B) PE-labeled CD63 or (C) CD107a (LAMP-1) antibodies were added to permeabilized cells to detect exosome-associated proteins. The majority of T cells were positive for CD63 and the increased mean fluorescence intensity (MFI) of SLE T cells was significant compared to HC (p = 0.027). All monocytes and neutrophils were positive for CD63 and CD107a and the increased MFI expressed of SLE monocytes was significant for both CD63 (p = 0.004) and CD107a (p = 0.047) as compared to HC.
(TIF)

Figure S6 **Elevated levels of thioredoxin and galectin-3 in SLE plasma**. Plasma levels of (A) thioredoxin and (B) galectin-3 were significantly elevated in SLE samples as compared to healthy control (HC). Protein levels were measured by routine ELISA as described in the methods.
(TIF)

Table S1 **List of the CD19^+^ B lymphocyte transcripts in the cluster order in which they appear in the** **figure 1** **heatmap.** Also shown are the fold change expression values for the inactive SLE and active SLE groups as compared to the HC group, Entrez gene name identifier, protein product cellular location and protein product molecular function for each transcript.
(XLS)

Table S2 **List of the CD3^+^ CD4^+^ T lymphocyte transcripts in the cluster order in which they appear in the** **figure 2** **heatmap.** Also shown are the fold change expression values for the inactive SLE and active SLE groups as compared to the HC group, Entrez gene name identifier, protein product cellular location and protein product molecular function for each transcript.
(XLS)

Table S3 **List of the CD33^+^ myeloid cell transcripts in the cluster order in which they appear in the** **figure 3** **heatmap.** Also shown are the fold change expression values for the inactive SLE and active SLE groups as compared to the HC group, Entrez gene name identifier, protein product cellular location and protein product molecular function for each transcript.
(XLS)
