# Supplementary figures and images for: SLE Peripheral Blood B Cell, T Cell and Myeloid Cell Transcriptomes Display Unique Profiles and Each Subset Contributes to the Interferon Signature
Source: PLoS One. 2013 Jun 24;8(6):e67003. doi: 10.1371/journal.pone.0067003 (PMC3691135; doi:10.1371/journal.pone.0067003)

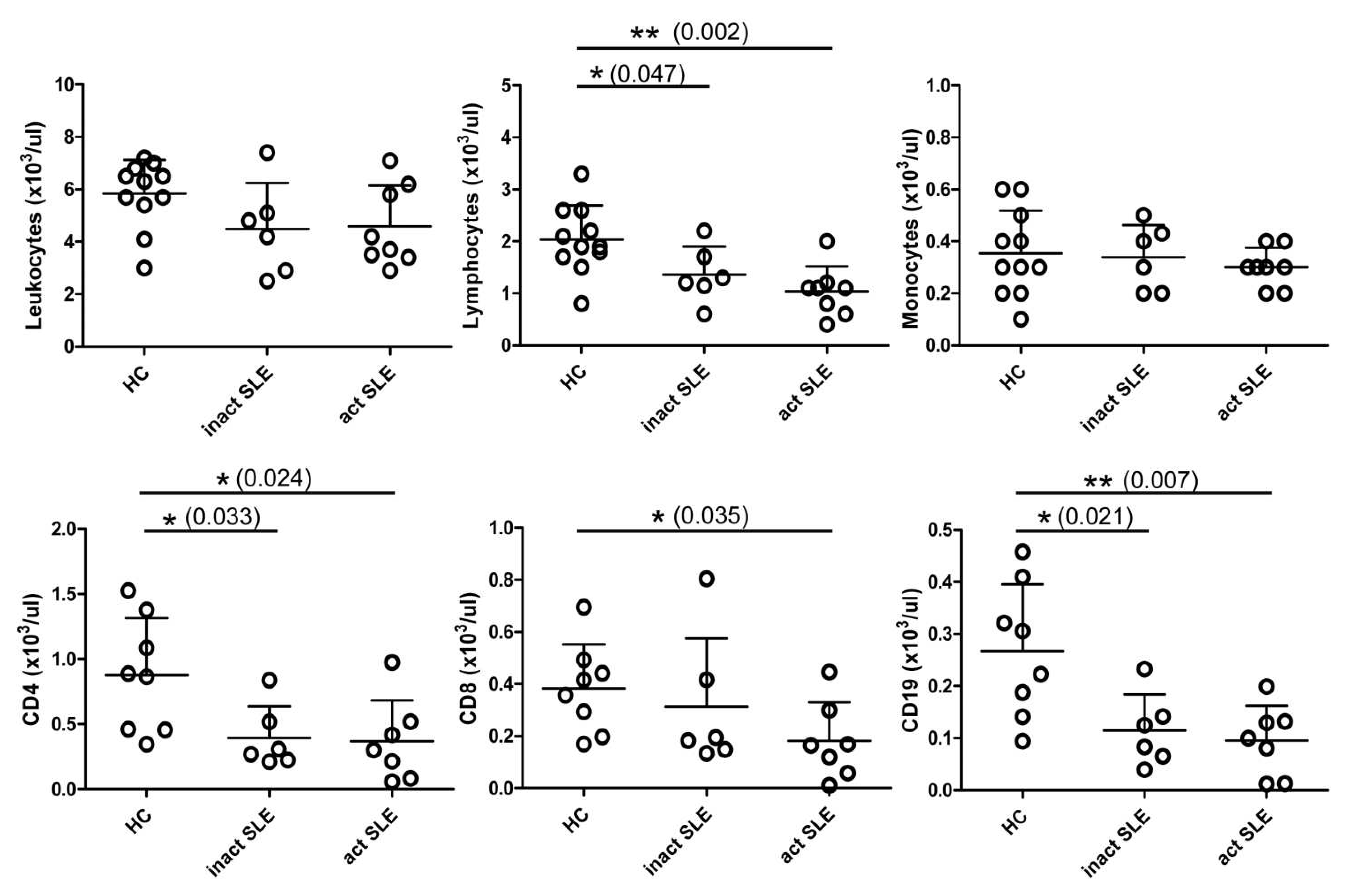

Supplement: Figure S1 — Leukocyte subset distribution in peripheral blood. (Upper panel) Cell counts and differentials were performed on blood samples collected for microarrays. Leukocyte, lymphocyte and monocyte counts are shown for healthy controls (HC) and inactive (SLEDAI ≤7) or active (SLEDAI ≥ 8) SLE samples. (Lower panel) Absolute numbers of lymphocytes were calculated from the cell count multiplied by the frequency of each subset. The frequency of HC and SLE lymphocytes were: CD3+CD4+ T cells (HC, 42% ±9%; median ± SD, N = 8 versus SLE, 30% ±12%; N = 13); CD3+CD8+ T cells (HC 19% ±5%, N = 8 versus SLE 20% ±7%, N = 12); CD3−CD19+ B cells (HC, 12% ±3%, N = 8 versus SLE 7% ±5%, N = 13). Frequencies of neutrophils, lymphocytes and monocytes in HC versus SLE were: neutrophils (54.7% ±9.9 vs 63.5% ±13.6; mean ± SD), lymphocytes (35.1% ±9.4 vs 26. 9±11.3) and monocytes (6.0% ±1.7 vs 7.1±1.5). Frequencies for SLE groups with inactive versus active disease were: neutrophils (57.6% ±15.4 vs 67.9% ±10.9), lymphocytes (31.6% ±13.1 vs 23.4±9.0) and monocytes (7.7% ±1.1 vs 6.7±1.6). These relative subset differences attained statistical significance when active SLE was compared to HC for neutrophils (p<0.05) and lymphocytes (p<0.05). Absolute numbers of SLE lymphocytes were decreased in inactive (p<0.05) and active disease (p<0.005) compared to HC. Absolute numbers of CD19+CD3− B cells and CD3+CD4+ T cells were significantly decreased in SLE patients (lower panel) and CD3+CD8+ T cell numbers were lower in patients with active disease. There was an increased frequency of SLE double negative lymphocytes (40.9% ±19.32, N = 12) as compared HC (28.27% ±12.95, N = 8). The greatest differences were observed in the relative frequencies of neutrophils and monocytes versus lymphocytes. (TIF) [file pone.0067003.s001.tif]

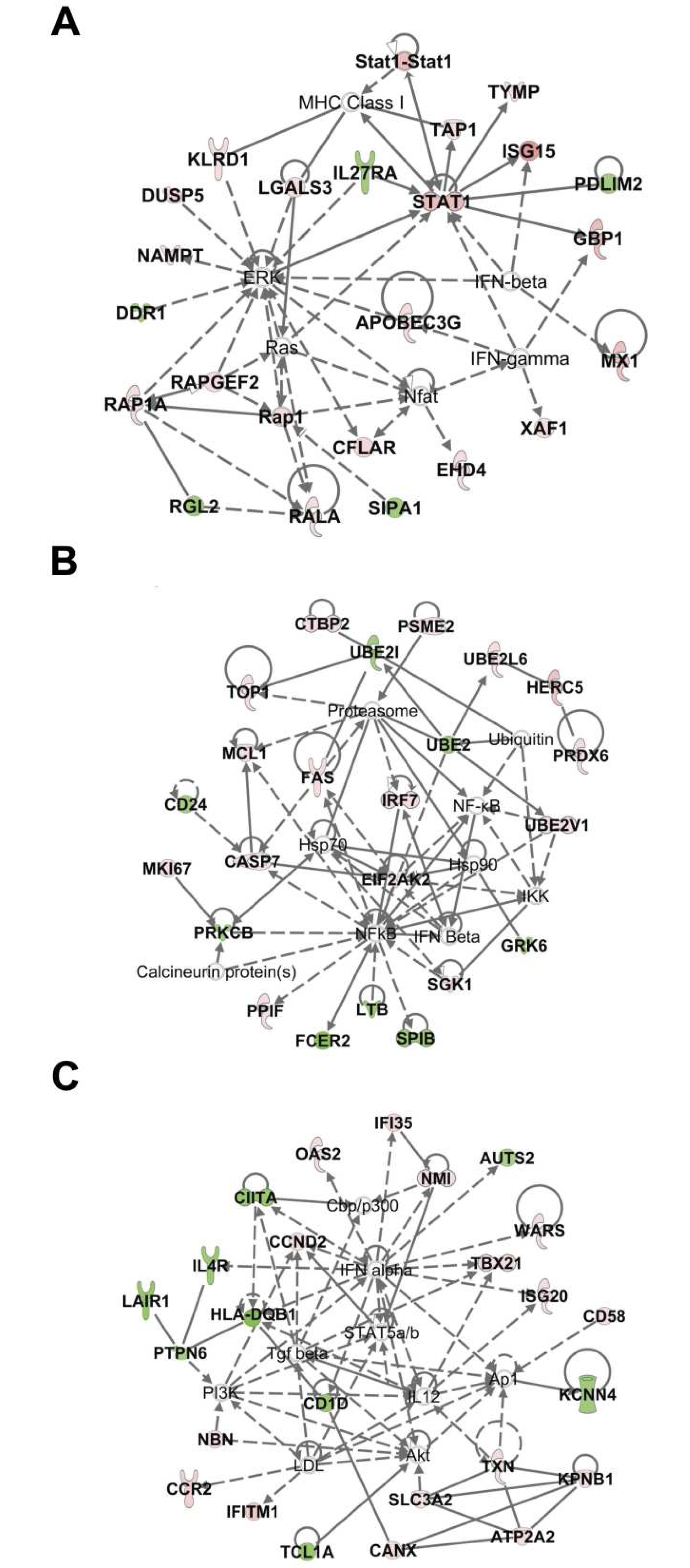

Supplement: Figure S2 — Network analysis of differentially expressed transcripts in SLE B cells. Differentially expressed transcripts in SLE B cells were tested for inter-relationships based on the published literature using IPA to obtain networks (A–C).The up-regulated expressed genes, relative to HC B cells are red with increasing intensity corresponding to increasing fold change and down-regulated expressed genes are shown in green. Direct interactions (binding or direct regulation) between products of transcripts are shown with solid lines and indirect relationships are shown using interrupted lines, as deduced from the published literature. (TIF) [file pone.0067003.s002.tif]

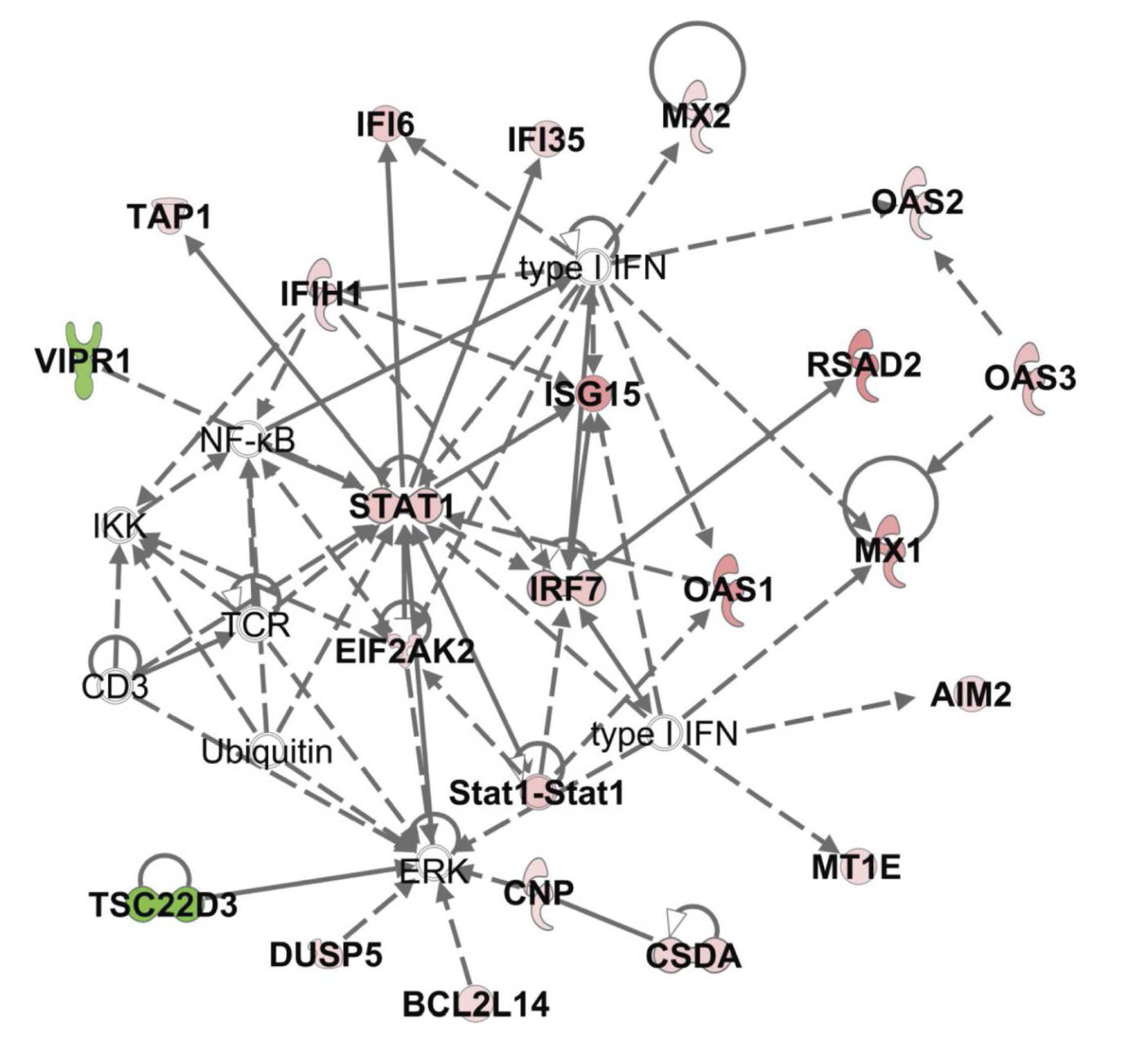

Supplement: Figure S3 — Network analysis of differentially expressed transcripts in SLE CD4+ T cells. Differentially expressed transcripts in SLE CD4+ T cells were tested for inter-relationships based on the published literature, using IPA as described in Figure S2. (TIF) [file pone.0067003.s003.tif]

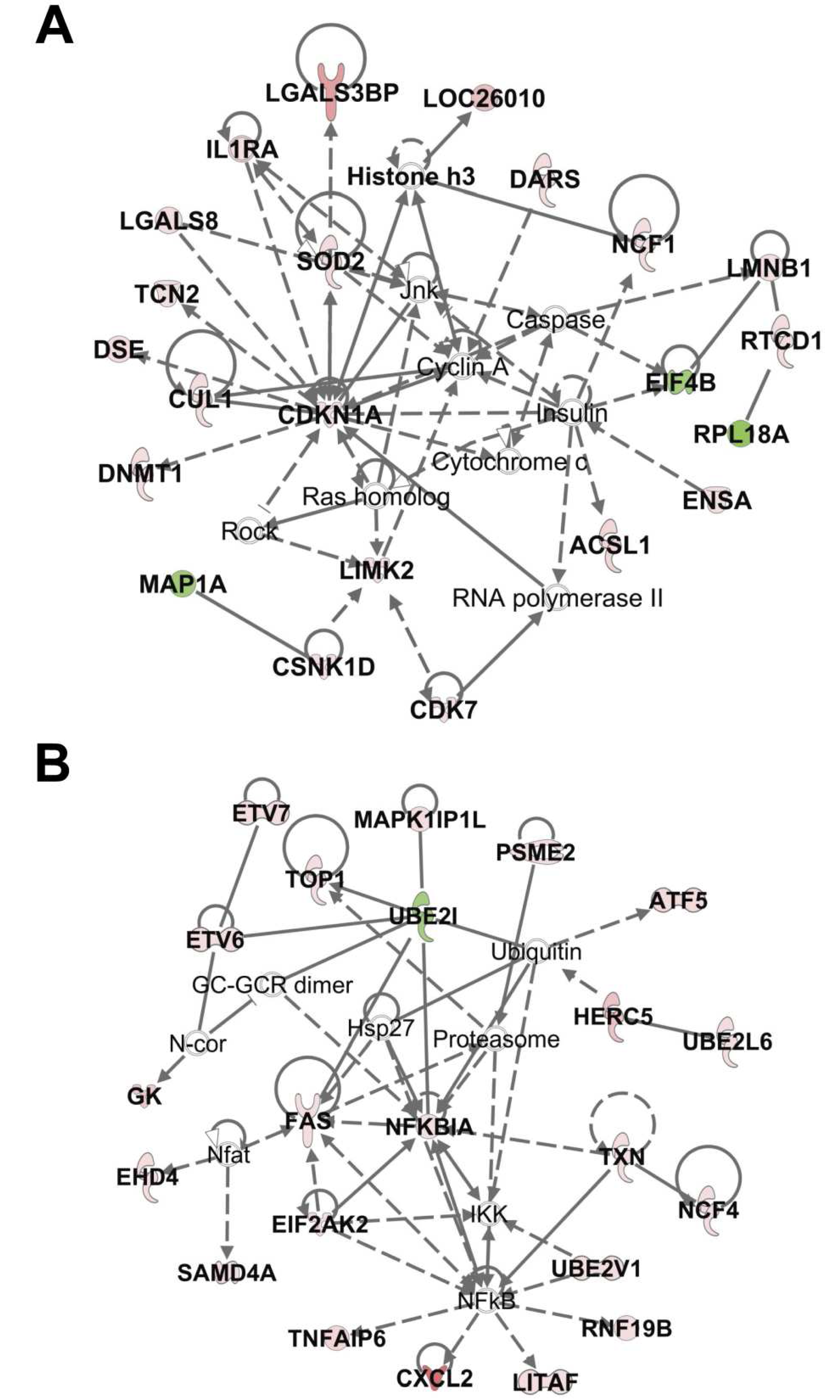

Supplement: Figure S4 — Network analysis of differentially expressed genes in SLE myeloid cells. Differentially expressed transcripts in SLE myeloid cells were tested for inter-relationships based on the published literature. Networks (A and B) assessed with IPA as described in Figure S2. (TIF) [file pone.0067003.s004.tif]

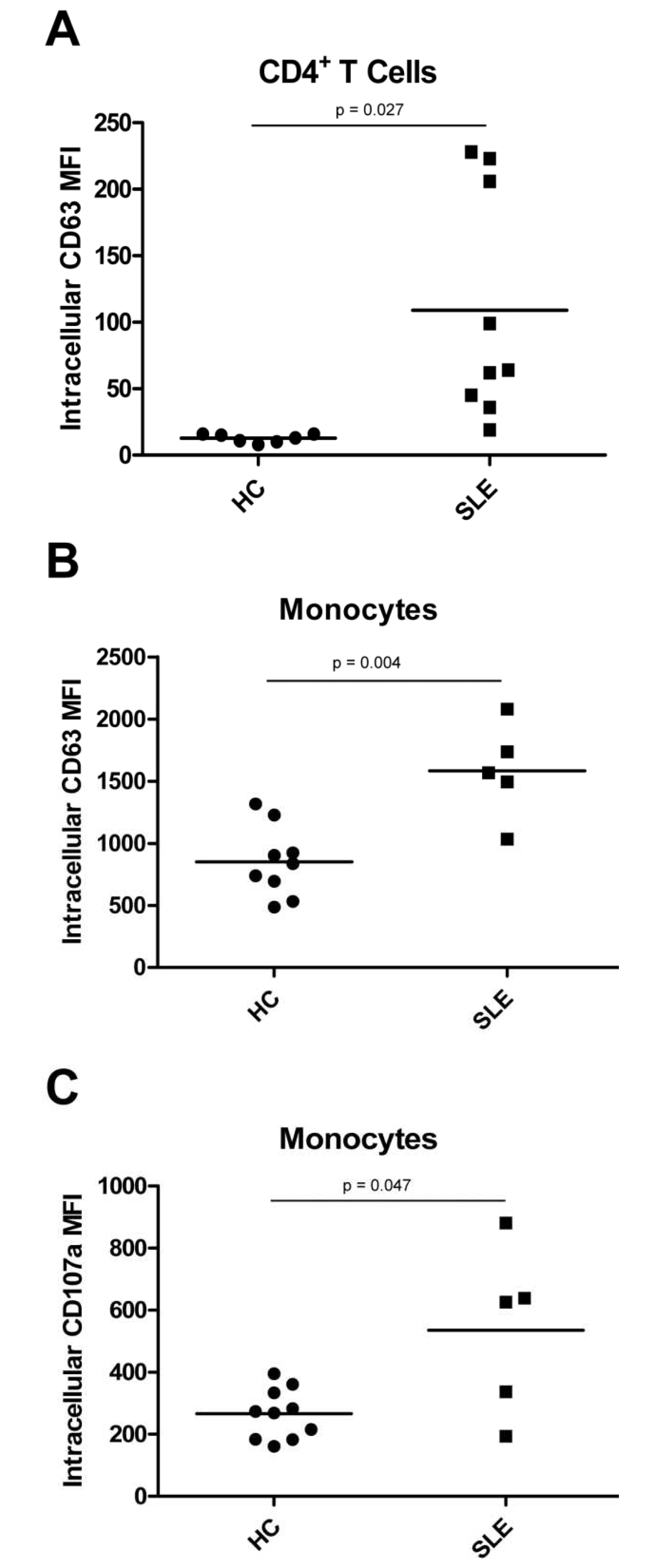

Supplement: Figure S5 — Elevated Endosomal Proteins in SLE T Cells and Myeloid Cells. Anti-CD3 and anti-CD4 antibodies were employed to identify CD4+ T cells. Anti-CD14 and/or anti-CD33 antibodies were utilized to identify monocytes. (A,B) PE-labeled CD63 or (C) CD107a (LAMP-1) antibodies were added to permeabilized cells to detect exosome-associated proteins. The majority of T cells were positive for CD63 and the increased mean fluorescence intensity (MFI) of SLE T cells was significant compared to HC (p = 0.027). All monocytes and neutrophils were positive for CD63 and CD107a and the increased MFI expressed of SLE monocytes was significant for both CD63 (p = 0.004) and CD107a (p = 0.047) as compared to HC. (TIF) [file pone.0067003.s005.tif]

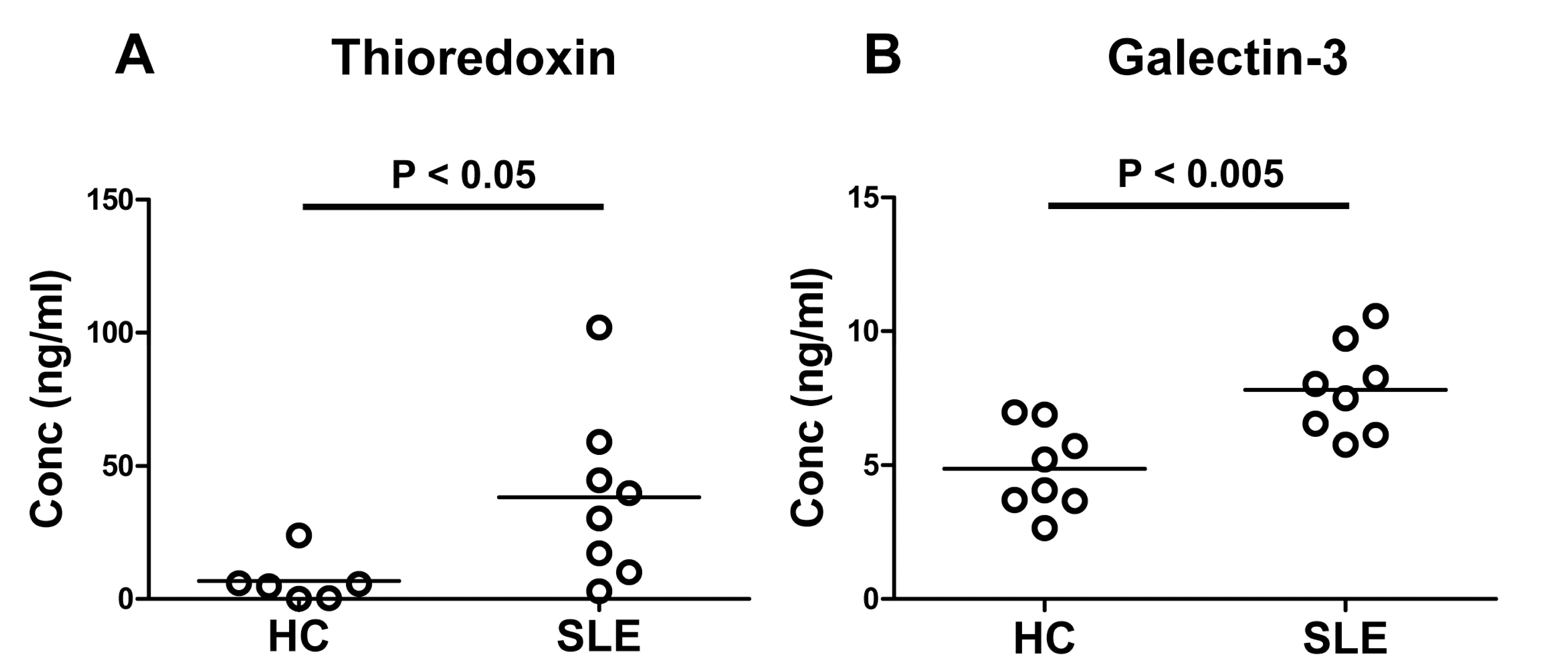

Supplement: Figure S6 — Elevated levels of thioredoxin and galectin-3 in SLE plasma. Plasma levels of (A) thioredoxin and (B) galectin-3 were significantly elevated in SLE samples as compared to healthy control (HC). Protein levels were measured by routine ELISA as described in the methods. (TIF) [file pone.0067003.s006.tif]
